# The Global Fund's resource allocation decisions for HIV programmes: addressing those in need

**DOI:** 10.1186/1758-2652-14-51

**Published:** 2011-10-26

**Authors:** Olga Avdeeva, Jeffrey V Lazarus, Mohamed Abdel Aziz, Rifat Atun

**Affiliations:** 1 The Global Fund to Fight AIDS, Tuberculosis and Malaria, Chemin de Blandonnet 8, CH-1214 Vernier, Geneva, Switzerland; 2 Global Fund to Fight AIDS, TB and Malaria and Copenhagen HIV Programme, Copenhagen University, Blegdamsvej 3B, DK-2200 Copenhagen N, Denmark; 3 Stop TB, East Mediterranean Regional Office, World Health Organization, Abdul Razzak Al Sanhouri Street, P.O. Box 7608, Nasr City, Cairo 11371, Egypt; 4 Imperial College London, London SW7 2AZ, UK

## Abstract

**Background:**

Between 2002 and 2010, the Global Fund to Fight AIDS, Tuberculosis and Malaria's investment in HIV increased substantially to reach US$12 billion. We assessed how the Global Fund's investments in HIV programmes were targeted to key populations in relation to disease burden and national income.

**Methods:**

We conducted an assessment of the funding approved by the Global Fund Board for HIV programmes in Rounds 1-10 (2002-2010) in 145 countries. We used the UNAIDS National AIDS Spending Assessment framework to analyze the Global Fund investments in HIV programmes by HIV spending category and type of epidemic. We examined funding per capita and its likely predictors (HIV adult prevalence, HIV prevalence in most-at-risk populations and gross national income per capita) using stepwise backward regression analysis.

**Results:**

About 52% ($6.1 billion) of the cumulative Global Fund HIV funding was targeted to low- and low-middle-income countries. Around 56% of the total ($6.6 billion) was channelled to countries in sub-Saharan Africa. The majority of funds were for HIV treatment (36%; $4.3 billion) and prevention (29%; $3.5 billion), followed by health systems and community systems strengthening and programme management (22%; $2.6 billion), enabling environment (7%; $0.9 billion) and other activities. The Global Fund investment by country was positively correlated with national adult HIV prevalence. About 10% ($0.4 billion) of the cumulative HIV resources for prevention targeted most-at-risk populations.

**Conclusions:**

There has been a sustained scale up of the Global Fund's HIV support. Funding has targeted the countries and populations with higher HIV burden and lower income. Prevention in most-at-risk populations is not adequately prioritized in most of the recipient countries. The Global Fund Board has recently modified eligibility and prioritization criteria to better target most-at-risk populations in Round 10 and beyond. More guidance is being provided for Round 11 to strategically focus demand for Global Fund financing in the present resource-constrained environment.

## Background

The Global Fund to Fight AIDS, Tuberculosis and Malaria is a public-private partnership dedicated to attracting and disbursing resources to address HIV, tuberculosis (TB) and malaria pandemics. As of the end of 2010, the Global Fund had allocated US$12 billion and disbursed $7.4 billion for HIV programmes, making it one of the leading sources of funding for HIV programmes worldwide. The resources from the Global Fund, along with resources from key partners, such as the US President's Emergency Plan for AIDS Relief (PEPFAR) and the World Bank Multi-Country HIV/AIDS Program, have made a major contribution to efforts to achieve universal access to prevention, treatment and care services for HIV and AIDS.

By 2009, the joint efforts in this significant expansion in resources had resulted in the reduction of new infections by 19% from the levels in 1999 [1]. However, the global population of people living with HIV continues to be large, numbering an estimated 33.3 million at the end of 2009 [1]. Sub-Saharan Africa remains the region most heavily affected by HIV, accounting for 68% of HIV infections worldwide. The Asian region is home to 4.9 million people living with HIV [2]. The Asian epidemic is still concentrated within specific high-risk populations. Nevertheless, with such a large population, just a small increase could have catastrophic effects [3].

The three regions of the Middle East and North Africa, Latin America and the Caribbean, and Eastern Europe and Central Asia also experience concentrated epidemics. HIV has more heavily affected the Caribbean Region than any other region outside sub-Saharan Africa, with the second highest adult prevalence in the world. In the Eastern Europe and Central Asia region, where injecting drug use is the primary mode of transmission, treatment levels are lower than in sub-Saharan Africa [2], and most people are unaware of their status.

The global economic recession is straining budgets in many low- and middle-income countries, with a decline in health overseas development aid, including commitments to the Global Fund [3]. The Third Voluntary Replenishment of the Global Fund, which led to pledges of US$11.7 billion, will enable further scale up of Global Fund investments for the 2011 to 2013 period, but not at the same pace as in recent years and it is insufficient to meet the anticipated demand. Therefore, not only is there a need to mobilize domestic resources and external aid for HIV programmes, but it is also necessary to ensure that available resources are used as efficiently as possible, and that allocation for HIV prevention, treatment, care and support services matches epidemiological patterns in order to maximize positive outcomes.

This study reviews the Global Fund HIV portfolio in 2002-2010 (funding rounds 1-10). It describes the trends and allocation patterns of the Global Fund investment in HIV programmes and assesses how these investments were allocated in relation to disease burden in the general population and among vulnerable groups, as well as to levels of national income.

## Methods

### Conceptual framework

The conceptual framework for this assessment is an analysis of funding flows and resource allocation patterns, using the National AIDS Spending Assessment (NASA) framework [4,5], developed by the Joint United Nations Programme on AIDS (UNAIDS). NASA allows for the monitoring of the annual flow of funds used to finance the response to HIV and AIDS. Its methodology is based on existing accounting approaches and the National Health Accounts framework [6], an internationally recognized tool for tracking financial flows on overall healthcare from funding sources to financing agents, service providers, services and beneficiaries.

The study presents the annual Global Fund-approved funding for HIV programmes by country, region of the world, epidemic type and spending category. Approved funding for the Global Fund HIV programmes is presented using NASA spending categories [4]: (1) prevention (including communication for social and behaviour change, counselling and testing, condom social marketing, and prevention of mother to child transmission); (2) care and treatment (including antiretroviral therapy, treatment of opportunistic infections, and collaborative TB/HIV activities); (3) interventions targeting orphans and vulnerable children; (4) programme management and administration (including planning, coordination, monitoring and evaluation, and operational research); (5) human resources (including workforce services on training, recruitment, retention, and rewarding of performance of the workforce involved in the HIV field); and (6) enabling environment (including advocacy, reduction of stigma and discrimination, and capacity building).

Using the NASA framework, the study analyzes the Global Fund flow of HIV investment from the Global Fund as the funding source, to interventions/spending categories and beneficiary populations.

### Methodology

We examined Global Fund-approved funding for HIV programmes in 2002-2010 (Rounds 1-10) in 145 countries for Phase 1 and 2 grants, exceptional extension funding, and funding provided through the Rolling Continuation Channel and National Strategy Application grants.

We collected data on the Global Fund-approved funding by spending categories from the proposal budgets, including for Rolling Continuation Channel proposals and National Strategy Application proposals, approved by the Global Fund Board as of the end of 2010 [7]. If the country grant proposal budget lacked detailed information about the allocation by service delivery area or if the amounts requested by the Country Coordinating Mechanism deviated after the Technical Review Panel review and Board approval, we used estimation methods to generate a complete dataset of approved funding disaggregated by spending categories.

If the proposal budget deviated from the Board-approved grant amount (difference less or equal to 10%), we assumed that the "error" (the difference between the proposal and the Board-approved budget) was proportionate across all spending categories. In such cases, we adjusted the original budget accordingly (for example, proportionate reduction by 10%). In other cases, a closely related expenditure figure served as a proxy [8,9]. The estimations for incomplete or deviated data were made based on the assumption that allocation pattern of expenditure (in the absence of any major reprogramming of Global Fund grants between 2002 and 2010) followed the allocation patterns of the grant-approved funding.

The amounts under consideration were distributed using proxy variables (we called them "allocation keys") as indicators of the likely distribution. For 2002-2010, the estimations were made for 131 (20%) out of 651 reviewed proposal documents. The estimations for incomplete data were made based on a review of national programmes, UNAIDS-reported data [10], HIV sub-accounts and NASA reports available for selected countries [11], the Global Fund Five-Year Evaluation database [12], and previous analyses of the Global Fund's portfolio [13]. In most of the cases, the budget proposals for early rounds (1-3) had a missing or incomplete breakdown by spending category that would bias one of the key findings of the study, such as resource allocation for most-at-risk populations. However, most of the Global Fund support for these populations was allocated through Rounds 8-10 and renewed grants that have reliable budget data in the proposal documents.

The UNAIDS definitions of HIV-related interventions were used to aggregate multiple interventions used in the country proposal budgets into a set of standardized NASA classification schemes. Proposal analysis allowed us to employ a bottom-up approach to calculate the total amounts of funds for all spending categories by country, funding round and year. The funding units (funding per spending category) from the proposals were aggregated to the level of funding per country and programme.

The estimated funding units were compiled into a single dataset for analysis. All results are presented in 2008 US dollars. Several important characteristics of countries and/or regions were assessed by:

• The type of epidemic (generalized, concentrated, low level) [1]

• Income levels of countries according to their 2009 gross national income (GNI) per capita using the World Bank Atlas method as per current Global Fund income eligibility criteria [14,15]

• Adult HIV prevalence and prevalence in most-at-risk populations (MARPs) [2,10].

We examined the Global Fund-approved funding per capita and its likely predictors, such as HIV adult prevalence, HIV prevalence in MARPs and GNI per capita as based on the current Global Fund income eligibility criteria [15]. Analysis was carried out using stepwise backward regression analysis. Details on the variables and the data sources are presented in Table 1. There were 140 countries included in the analysis. Analysis was done in SPSS (version 18.0).

**Table 1:** Variable definitions and data sources

Variable	Definition	Source of data
National HIV adult prevalence	The percentage of estimated number of all adults 15-49 living with HIV in the country, divided by population in 2002-2009	UNAIDS [1,10]

HIV prevalence in most-at-risk populations	The percentage of people who inject drugs, sex workers, and men who have sex with men who are HIV positive in the country, divided by the population in 2002-2009	UNAIDS [1,10]

Gross national income per capita	The gross national income, converted to US dollars using the World Bank Atlas method, divided by the mid-year population	World Bank [14]

The Global Fund annual median funding per capita	The median Global Fund approved funding per country per year converted in 2008 US dollars divided by mid-year population	Estimates of the study

## Results

By the end of 2010, the Global Fund had approved US$12 billion for HIV programmes in 145 countries. The level of annual HIV investment expanded from $0.3 billion in 2002, when the Global Fund was established, to $1.1 billion in 2003, $2.0 billion in 2008, $2.5 billion in 2009 and $1.2 billion in 2010.

Of the eight Global Fund regions, the three sub-Saharan Africa regions showed the highest absolute gain in investments over time, especially after the high rates of approved funding in Round 8, increasing from US$0.2 billion in 2002 to $1.2 billion in 2008 and $1.1 in 2010), while the Middle East and North Africa region saw the greatest percentage increase. Other regions demonstrated a steady scale up during the reporting period, displaying the highest increases in Rounds 8 and 9.

### Allocation of the Global Fund-approved HIV funding by spending categories

**Figure 1:**
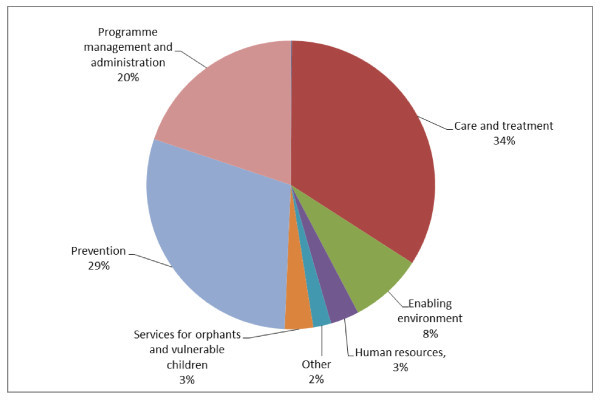
**The Global Fund allocations by spending categories: cumulative portfolio, 2002-2010**. Source: The Global Fund grant portfolio database [7].

In 2002-2010, most of the funds were allocated to care and treatment ($4.3 billion or 36%) and prevention ($3.5 billion or 29%), followed by health systems and community systems strengthening and programme management and administration ($2.6 billion or 22%) (Figure 1). Funding of US$0.9 billion, or 7%, was approved for ensuring an enabling environment in countries. Funding for services aimed at improving the lives of orphans and other vulnerable children affected by HIV accounted for $0.3 billion or 3% of the cumulative funding. About 3% or $0.3 billion was approved for workforce activities targeting retention, deployment and rewarding of personnel working in the HIV programmes. The remaining funds were allocated to activities that were classified as "other".

**Figure 2:**
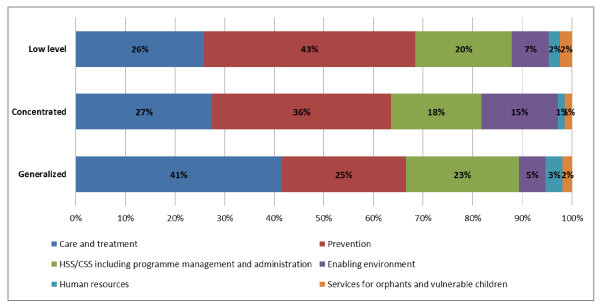
**Allocation of the Global Fund approved funding by type of epidemics**. Source: The Global Fund grant portfolio database [7].

In 2002-2010, the Global Fund allocated the majority of its HIV funding to countries experiencing generalized epidemics (US$8 billion or 68%). Countries with generalized epidemics received the highest median per capita funding ($2.9). Funding is allocated to a lesser extent to countries with concentrated epidemics ($2.9 billion or 25% of the total portfolio and $1.2 per capita) and low-level epidemics ($0.9 billion or 7% of the total portfolio and $1.0 per capita). The Global Fund resource allocation to specific programmes addressing HIV prevention, care and treatment and non-health categories varies among countries with different types of epidemics, as presented in Figure 2. Overall, countries with low-level and concentrated epidemics allocate a higher proportion of their funds to prevention (43% and 36%, respectively), while countries with generalized epidemics allocate a larger share to care and treatment (41%).

In the countries with concentrated epidemics driven by sexual and injecting drug practices among at-risk groups, interventions focusing on an enabling environment account for a larger share (15%) as compared with countries with other types of epidemics. These interventions primarily focus on improving the environment for safer sex work, as well as stigma reduction.

**Figure 3:**
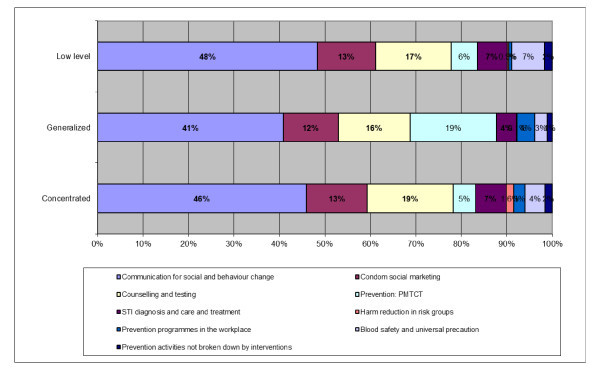
**Allocation of the Global Fund approved funding for prevention by type of epidemics**. Source: The Global Fund grant portfolio database [7].

The overall allocation of the Global Fund resources for prevention varies significantly by type of epidemic. Figure 3 presents allocation of funding by type of epidemic. In all epidemiological settings, countries showed a tendency to prioritize interventions for behaviour change communication (BCC). BCC accounted for 38% to 54% of the cumulative prevention funding. Around 12% was allocated for condom distribution, and 14% to 16% to counselling and testing in all epidemiological settings. Funding for prevention of mother to child transmission services was higher, at 20%, in countries experiencing generalized epidemics as compared with the other types of epidemics, where it received only 5% to 6% of the cumulative prevention funding.

### The Global Fund investment addressing most-at-risk populations

A separate analysis was conducted on HIV resources allocated to specific risk groups, in particular for programmes targeting people who inject drugs, sex workers and men who have sex with men (MSM). Cumulatively approved funding addressing HIV prevention in these risk groups through HIV programmes represented US$349 million or about 10% of funding on HIV prevention in 2002-2010 as compared with 6% of the cumulative funding till Round 10.

**Figure 4:**
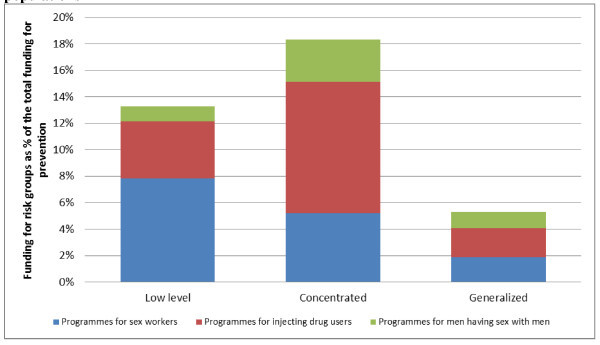
**Allocation of the Global Fund cumulative approved funding for most-at-risk populations**. Source: The Global Fund grant portfolio database [7].

Figure 4 presents the allocation of the Global Fund-approved funding for people who inject drugs, MSM and sex workers by type of epidemic. The highest share, 18% of HIV prevention funding, targeted these three groups in countries with concentrated epidemics with the rest of the prevention funds invested in interventions for the general population. In the countries with generalized epidemics, funding for these risk groups accounted for 5%; in the countries with low-level epidemics, it represented 13% of cumulative funding for HIV prevention. The remaining prevention funds were allocated for interventions targeting the general population. Relatively low levels of funding were allocated to the prevention interventions targeting MSM ($63 million or 2% of total prevention funding for MARPs), even in countries with concentrated epidemics.

Most of the funding for MARPs was channelled through BCC interventions. Cumulatively, in 2002-2010, the Global Fund invested $1.5 billion in HIV BCC interventions. About 13% of these funds, or $199 million, was allocated for BCC for most-at risk populations.

During the reporting period, the Global Fund cumulatively invested $392 million in condom distribution programmes. The condom distribution programmes for MARPs accounted for 13% of the total, or $52 million.

### Allocation in accordance with health needs and national income

The median annual funding per capita for Global Fund-supported HIV programmes was compared with the countries' disease burdens, measured as the share of adult HIV prevalence and prevalence among MARPs. The Global Fund funding per capita was also compared with the level of GNI per capita.

The majority of Global Fund funding for HIV programmes (52%) and the highest median annual per capita funding ($2.3) was allocated to low-income countries; 34% of HIV funding ($1.3 per capita) was allocated to lower-middle income countries; while 14% ($1.1 per capita) was allocated to upper-middle income countries.

Forty-three low-income countries received 52% of cumulative funding for HIV programmes from the Global Fund, while 55 lower-middle-income and 42 upper-middle-income countries jointly accounted for 48% of cumulative Global Fund support for HIV. Several countries with different levels of income (upper-middle-income and low-income) receive similar funding per capita regardless of their GNI level. Upper-middle-income countries, such as Croatia, Mexico and the Russian Federation, received per capita funding (less than US$1) from the Global Fund, comparable with low-income countries like Bangladesh and Madagascar.

We next assessed the likely predictors of the Global Fund resource allocation to HIV programmes in 2002-2010 (Rounds 1-10). The predictor variables were selected based on the Global Fund country eligibility criteria for funding that take into consideration GNI per capita, adult HIV prevalence and the prevalence of HIV in MARPs. Table 2 presents the predictors of Global Fund funding per capita. The coefficients of the regression show a more significant effect of adult HIV prevalence and MARPs prevalence on funding per capita in all 145 countries with approved HIV grants. These results were consistent for sub-group analysis for low-income and upper-middle-income countries and for the regional sub-analysis presented in Table 2.

**Table 2:** Assessing the predictors of Global Fund funding per capita

Variables	GNI per capita, 2009	HIV prevalence 14-45, 2009	Prevalence in MARPs
**All countries-recipients of the Global Fund HIV programmes (n = 145)**			

Annual median per capita funding for HIV	0.282 (1.415)*	0.313 (1.937)***	0.370 (2.118)***

**Low-income countries (n = 40)**			

Annual median per capita funding for HIV	NS	0.483 (1.795)**	0.338 (-0.047)*

**Upper-middle-income countries (n = 37)**			

Annual median per capita funding for HIV	NS	0.425 (1.380)***	0.820 (2.412)***

**Concentrated epidemics (n = 52)**			

Annual median per capita funding for HIV	0.121 (2.244)**	0.311 (1.840)*	0.580 (1.205)*

**Generalized epidemics (n = 48)**			

Annual median per capita funding for HIV	0.427 (2.467)***	0.250 (1.322)**	0.480 (1.783)**

**Sub-Saharan Africa region (n = 43)**			

Annual median per capita funding for HIV	NS	0.355 (2.073)	0.118 (0.959)

**Eastern Europe and Central Asia region (n = 24)**			

Annual median per capita funding for HIV	0.621 (-1.446)*	0.430 (2.104)**	0.625 (2.1943)***

**Latin America and Caribbean region (n = 30)**			

Annual median per capita funding for HIV	NS	0.530 (1.775)*	0.748 (2.430)***

**Asia region (n = 27)**			

Annual median per capita funding for HIV	NS	0.350 (1.840)	0.348 (1.271)

Results of the analysis for the coefficient of the GNI per capita showed no strong effect on the per capita funding for HIV (0.165, significant at p < 0.05). However, sub-analysis by type of epidemics showed a strong positive effect of GNI per capita in countries with generalized epidemics. Regional sub-analysis revealed a positive effect of GNI per capita on the Global Fund investment only in the Eastern Europe and Central Asia region.

## Discussion

The Global Fund's guiding principles target investments in line with need for HIV, tuberculosis and malaria, and enable allocation of funding based on country demand.

The key HIV funding provided by the Global Fund was for HIV treatment and care (35%) and prevention activities (29%). There is an emerging consensus that appropriately targeted "know-your-epidemic" prevention efforts need to be expanded and the mix between treatment and prevention interventions need to be adjusted according to the national epidemiological context and assessment of the roots of HIV transmission in the country. In contrast, earlier start points (CD4 cell count of 350 cells/mm^3^), improved treatment regimens, more effective linkages to care and adherence support and the treatment-as-prevention paradigm [16,17] would all increase investments needed for HIV treatment and care.

Differences in allocation patterns were observed in relation to the dynamics and severity of the epidemics. The majority of the Global Fund HIV investments (69% of cumulative funds) and the highest per capita funding were channelled to countries in sub-Saharan Africa experiencing generalized epidemics. These countries allocated about 40% of their funding for HIV care and treatment activities. The review of the investment of other key donors in HIV control showed that in 2002-2009, most PEPFAR funds also went to countries with generalized epidemics and mostly for HIV treatment [18], whereas domestic and international funding for prevention remained underfunded [19]. Global investment into HIV treatment and prevention could bring better outcomes if national and international efforts to control HIV epidemics were balanced between the most effective programmatic interventions.

A lower share of the Global Fund HIV investment, as well as lower per capita funding, was targeted to countries experiencing concentrated and low-level epidemics where the recorded infection was largely confined to individuals with risk behaviours, for example, sex workers, people who inject drugs and men who have sex with men. Our analysis showed variability in the Global Fund funding for prevention interventions by type of epidemics. All Global Fund countries prioritized behaviour change communication interventions in their prevention activities, reaching about half of all prevention funds in countries with low-level epidemics. However, cumulatively, only 11% of all of such interventions targeted most-at-risk populations, which are more effective in settings where HIV burden is high among risk groups [20-24].

The next priority for Global Fund recipients was social marketing of condoms and HIV counselling and testing. While there is some evidence of success in turning around generalized HIV epidemics by changing sexual behaviour, this turns out to be most effective in risk groups in concentrated epidemics [25-29]. Several studies show only modest evidence for the effectiveness of counselling and testing activities in generalized epidemics settings compared with concentrated epidemics, but concluded that it should not negate the need to expand them [30-35]. Its great potential should be weighed against other interventions in allocating prevention funding.

In 2002-2010, about 10% of the Global Fund's cumulative approved funding for HIV prevention was allocated to interventions targeting sex workers, people who inject drugs and men who have sex with men. In countries with concentrated and low-level epidemics, funding for interventions targeting prevention in most-at-risk populations account for 18% and 13% of all prevention activities, respectively. The rest of the preventive funds were invested in interventions for the general population that did not address the epidemiological context of the concentrated epidemics. New evidence suggests that targeted approach in funding allocated to the major risks of transmission and acquisition of HIV infection in the concentrated epidemics provides the greatest effect and substantial changes might be possible with a few appropriately targeted efficacious interventions [36].

Although there was low funding for the most-at-risk populations, a review of the UNAIDS country reports on HIV financing in 2005-2009 showed that the Global Fund was the only or the major funding source targeting risk groups for HIV prevention activities for most-at-risk populations in many countries of the Eastern Europe and Central Asia region (such as Albania, Armenia, Bulgaria, Croatia, Georgia, Kazakhstan, Kyrgyzstan, the former Yugoslav Republic of Macedonia, Romania, Tajikistan and Ukraine), as well as in countries of other regions (such as Algeria, China, Ecuador, Madagascar, Mongolia, Swaziland and Thailand) [1,7,10,37].

The Global Fund resource allocation model seeks to ensure that funding is going to where it is most needed. For the purposes of this analysis, the need is interpreted in terms of HIV burden and national income [38]. The observed relationships between the HIV funding per capita, national HIV prevalence and prevalence in MARPs indicate that the Global Fund resource allocations to HIV programmes best correspond to the HIV prevalence in the applicant countries.

Our analysis shows that the Global Fund eligibility criteria resulted in allocating more funds to countries with lower national income. In 2002-2010, the Global Fund provided more support to low- and low-middle income countries (52% and 34% of cumulative funding and US$2.3 and $1.3 per capita, respectively), which is in line with the equity principles of the Global Fund [15]. Country GNI per capita, although positive, was not statistically significant with regards to the Global Fund allocations per capita, except for the Eastern Europe and Central Asia region and within the group of countries with generalized epidemics. For some upper-middle-income countries, mostly representing the Eastern Europe and Central Asia and the Latin America and Caribbean regions, the funding per capita was comparable to those in low-income countries, disregarding the higher cost of living and higher unit cost of HIV interventions in the concentrated HIV transmission settings of these regions. This demonstrates that the Global Fund invests in HIV programmes in countries with the least financial ability to address the problem.

However, within this group, the HIV funding does not linearly correspond to the country's national income. The national HIV prevalence and prevalence in MARPs predict the magnitude of the Global Fund investment, acknowledging the focus of the Global Fund programmes not only on the income level of the countries, but also in prioritizing the most-in-need countries and population groups; the latter was addressed in Round 10 (2010). A targeted response to concentrated epidemics is being achieved through revised prioritization criteria adopted by the Global Fund Board for Round 10 that allowed upper-middle-income countries to access funding solely for most-at-risk populations.

This expansion of the Global Fund eligibility criteria for upper-middle income countries allowed the organization to overcome one of the drawbacks of the use of the GNI per capita Atlas method indicator as one of the eligibility criteria as it is affected by annual fluctuations in the value of the respective domestic currencies in relation to the US dollar [39,40] and excludes some countries in need from being eligible to receive support from the Global Fund. The use of the GNI per capita indicator as a criteria for eligibility for Global Fund support does not account for the sub-national distribution of income, which is part of the social policy in many upper-middle-income applicant countries, where sub-national averages of income significantly deviate from national averages and affect subsequently equity in resource allocation by income [41-44]. The regression analysis we conducted using purchasing power parity did not bring significant difference in the results; thus, we are not presenting them in this paper. We have not adjusted our analysis to control for the variations in the unit cost of service delivery in the countries with different income level that might evidence a stronger correlation between GNI and the Global Fund funding.

This study assessed only some of the considerations that predict the Global Fund's funding decisions. These include HIV prevalence, prevalence of risk factors and national income. However, there are other factors that influence Global Fund resource allocation, as well as the country's demand for HIV funding, such as the potential for a rapid increase in burden of disease due to the current trends, size of population at risk, and extent of cross-border and internal migration.

The Global Fund resource allocation decisions are also based on the levels of national contributions to the financing of the proposal and contributions of other key funders, such as PEPFAR, the World Bank and the Bill & Melinda Gates Foundation, in order to ensure that Global Fund support for HIV is as additional to other sources as possible. The country capacity to implement the grant and existence of supportive national policies play a vital role in the distribution of the Global Fund's resources. These are the areas that should be further explored to ensure an evidence- and performance-based resource allocation for HIV control in the Global Fund recipient countries.

## Conclusions

The Global Fund resource allocation model allows for the scale up of investment in HIV prevention, treatment, care and support programmes, and its funding is aligned with HIV burden and national income. However, prevention in most-at-risk populations still does not have an urgent enough priority in most of the country programmes supported by the Global Fund. The intensified and targeted response to HIV control in these populations was further addressed through revised prioritization criteria adopted by the Global Fund Board for Round 10. More guidance is being provided for Round 11 to strategically focus demand for Global Fund financing, which is crucial in the present resource-constrained environment.

## Competing interests

During the manuscript writing all the authors worked for the Global Fund to Fight AIDS, Tuberculosis and Malaria.

## Authors' contributions

OA contributed to the conception and design of the study, data collection, analysis and its interpretation, as well as drafting of the initial manuscript. JVL made substantial contributions to data interpretation and revising of the manuscript. MAA was involved in the drafting of the manuscript and substantially contributed to data interpretation. RA substantially contributed to the conception and design of the study, as well as to data interpretation. All authors have read and approved the final manuscript.
